# Comparison of the immune effects of the *Chlamydia abortus* MOMP antigen displayed in different parts of bacterial ghosts

**DOI:** 10.3389/fmicb.2024.1349746

**Published:** 2024-02-07

**Authors:** Huaiyu Zhang, Zhaocai Li, Wei Li, Youshun Jin, Yunhui Li, Qian Xiao, Dewen Tong, Jizhang Zhou

**Affiliations:** ^1^State Key Laboratory for Animal Disease Control and Prevention, College of Veterinary Medicine, Lanzhou University, Lanzhou Veterinary Research Institute, Chinese Academy of Agricultural Sciences, Lanzhou, China; ^2^College of Veterinary Medicine, Northwest A&F University, Yangling, China; ^3^Gansu Province Research Center for Basic Disciplines of Pathogen Biology, Lanzhou, China

**Keywords:** bacterial ghosts, vaccine, delivery vector, *Chlamydia abortus*, MOMP antigen

## Abstract

Bacterial ghosts (BGs) are promising vaccine platforms owing to their high adjuvant properties and delivery efficiency. Heterologous antigens can be anchored to different parts of BGs using genetic engineering strategies to prepare vaccines. However, several key issues need to be resolved, including the efficient preparation of BGs and determining the optimal anchoring position of exogenous antigens in the BGs. Here, we prepared an efficient temperature-controlled lysis system using lysis gene E of phage PhiX174 and used the major outer membrane protein (MOMP) of *Chlamydia abortus* (*C. abortus*) as a model antigen to explore the optimal display location of exogenous antigens in BGs. We demonstrated that the constructed recombinant temperature-controlled lysis plasmid can still stably inhibit E gene expression at 37°C, and the lysis efficiency of *E. coli* can reach above 99.9%. Four recombinant MOMP *Escherichia coli* (*E. coli*) ghost vaccines were constructed using different anchor sequences. These vaccines all induced strong specific antibody responses and secrete high levels of IFN-γ in immunized mice and significantly increased the clearance of *C. abortus* in a mouse infection model. Notably, the strongest immune effect was observed when MOMP was displayed on the surface of *E. coli* ghosts (rECG-InpN-M), which resulted in the clearance of *C. abortus* in mice 6 days earlier than that with the recombinant MOMP vaccine. Altogether, we constructed an efficient BG temperature-controlled lysis system and provided a feasible strategy for developing a BG delivery platform with enhanced immune effects.

## 1 Introduction

*Chlamydia abortus* (*C. abortus*) is a pathogen that can cause ovine enzootic abortion in goats, sheep, pigs, and cattle, resulting in significant economic losses to the global livestock industry and posing a great public health safety risk (Longbottom et al., 2002). The infection of pregnant animals with *C. abortus* usually results in preterm, stillborn, or mummified fetuses, whereas the majority of other infected animals show asymptomatic or subclinical signs, which increases the risk of pathogen transmission between herds (Zhang et al., 2023). Therefore, safe and effective vaccines are the most efficient way to effectively control the infection. Although whole-cell-based inactivated vaccines have been developed, with, the conformation of the antigen is destroyed to some extent during the inactivation process, resulting in limited protection. In addition, the live attenuated 1B vaccine, although effective, is associated with the risk of virulence being restored. Moreover, these vaccines are expensive, and the production process involves safety risks (Pan et al., 2015). Therefore, subunit vaccines, with a low cost, good safety, and few side effects, have become an important direction for the development of *Chlamydia* vaccines in recent years.

Subunit vaccines often require efficient delivery systems to induce optimal protective immunity, and in this regard, the bacterial ghost (BG) platform has outstanding delivery advantages. BGs are a novel technology platform for inanimate vaccination, which are typically empty bacterial shells formed by the lysis of the cleaved protein E of phage PhiX174 (Ebensen et al., 2004). The most notable advantage of BGs over other abiotic vaccines is that they have an undamaged, intact cell wall structure and contain several Toll-like receptor agonists on their surface, allowing them to be efficiently recognized by antigen-presenting cells (APCs), which induce a strong immune response against the BGs or exogenous antigens delivered by the BGs (Ebensen et al., 2004; Won et al., 2020). In addition, BGs themselves act as natural immune adjuvants, and when an exogenous antigen is expressed in BGs, the antigen and adjuvant combine to become a single entity with an increased likelihood of uptake by the same APCs, further enhancing the immune effect (Chatzikleanthous et al., 2020). The BG delivery system retains many of the advantages of bacteria, including strong adjuvant properties, high delivery potential, the ability to colonize and target tissue cells, enhanced vaccine immunogenicity, and good loading capacity (Hajam et al., 2017). They are increasingly being used to deliver nucleic acids, proteins, and drugs (Paukner et al., 2004; Kudela et al., 2010; Langemann et al., 2010). Among these, genetic engineering methods for expressing exogenous antigens in BGs as subunit vaccine delivery carriers have gained considerable attention in research (Muhammad et al., 2012).

Four strategies have been used for the subcellular localization of antigens during the construction of recombinant BG vaccines: the inner membrane, outer membrane, cytoplasm, and periplasmic space (Chen et al., 2021). The spaces and environments of these four parts differ, therefore, the expression of antigens in these different regions has its own advantages. The target antigen can be anchored on the inner membrane of the BGs through the E’ or L’ sequence of the phage, and the activity of membrane-bound enzymes will not be affected by membrane binding (Jalava et al., 2003). Antigens can also be displayed on the surface of BGs by ice-nucleating proteins (Inp) or bacterial outer membrane proteins. There is sufficient space on the surface of BGs to favor proper antigen folding and facilitate processing by antigen-presenting cells (Bao et al., 2015; Gong et al., 2020). Because the bacterial S-layer protein self-assembles to form a sheet-like structure, inserting the antigen sequence into the S-layer protein sequence and expressing it together in the cytoplasm will not form inclusion bodies, and they will not be released into external media during the E protein cleavage process (Mayr et al., 2005). The target antigen can be expressed in the periplasmic space of bacteria after fusion with the maltose-binding protein (MBP). During cleavage of the E protein, the fusion of the inner and outer membranes tightly seals the periplasmic space, preventing leakage of the antigen. Moreover, the periplasmic space is rich in membrane-derived oligosaccharides that provide a stable environment for antigens to avoid inactivation (Riedmann et al., 2003).

Two central problems have been encountered in the development of BG-based subunit vaccines: firstly, ensuring that BGs are produced efficiently and conveniently; and secondly, determining the optimal anchoring position of heterologous antigens within BGs. Theoretically, when exogenous antigens are displayed on the surface of a delivery vehicle, they often induce stronger immune responses because they are easily recognized by the host immune system (Bermúdez-Humarán et al., 2004; Paukner et al., 2006). However, this may also be related to the quantity of antigen in the vector (Norton et al., 1996). It remains to be verified at which part of BGs the antigen can be anchored to produce the best immune effect.

The major outer membrane protein (MOMP) of *C. abortus* is a highly conserved antigen with good immunogenicity (Eko et al., 2004). Therefore, we chose the MOMP antigen to construct a vaccine using *Escherichia coli* (*E. coli*) ghosts (ECGs) as carriers to prevent *C. abortus* infection. The findings indicated that we constructed a recombinant temperature-controlled lysis plasmid that enabled efficient ECG preparation. This demonstrates that the best immune effect was achieved when MOMP was displayed on the surface of the ECG (rECG-InpN-M). In summary, this study provides a feasible strategy for developing recombinant BG vaccines and validates that ECGs are a reliable platform for adjuvant-free vaccine delivery.

## 2 Materials and methods

### 2.1 Ethics statement

Specific pathogen-free (SPF) female BALB/c mice aged 6 ~ 8 weeks were provided by the Laboratory Animal Center of the Lanzhou Veterinary Research Institute (Lanzhou, China) and bred in an SPF environment. The animals were maintained on a standard diet with *ad libitum* access to water. All animal experiments were reviewed and approved by the Animal Ethics Committee of the Lanzhou Veterinary Research Institute, Chinese Academy of Agricultural Sciences (No. LVRIAEC-2023-062), and followed the Animal Ethics Procedures and Guidelines of the People’s Republic of China.

### 2.2 Strains, cells, plasmids, and culture conditions

The cells, plasmids, and bacterial strains used in this study are listed in Supplementary Table 1. All *E. coli* strains were cultured aerobically in a standard lysogeny broth (LB) medium. Unless otherwise stated, *E. coli* containing pBV220-E or pUC-WK-E plasmids (see “Construction of Plasmids” below) were cultured at 30°C and 200 rpm/min. The other *E. coli* strains were cultured at 37°C and 200 rpm/min. The final concentrations of kanamycin, chloramphenicol, and ampicillin in LB were 100 μg/mL (Kan^+^), 50 μg/mL (Chl^+^), and 100 μg/mL (Amp^+^), respectively. The *C. abortus* GN6 strain (Li et al., 2015) was cultured on cells according to the method described by Onorini et al. (2019) and stored at −80°C.

### 2.3 Construction of plasmids

Supplementary Table 2 lists all polymerase chain reaction amplification and point mutation primers. Primers (E-F/E-R) were used to amplify the E gene of the phage in the pUC-E plasmid and were cloned into the pBV220 plasmid to construct the temperature-controlled cleavage plasmid pBV220-E. The temperature-controlled cleavage cassette “CIts857-λpR/pL-E-rrnB” of pBV220-E was amplified using primers (WK-E-F/WK-E-R), designated “WK-E,” and cloned into the pUC57 Kan plasmid to form the pUC-WK-E plasmid, which was lysed. The mutation primers (mut-F/mut-R) were designed as previously described (Jechlinger et al., 2005b) using the site-directed mutagenesis kit (Tiangen, China) to mutate the 32nd nucleotide of the λpR promoter of the temperature-controlled cleavage cassette WK-E from A to G and the 41st nucleotide from T to C. Consequently, the recombinant temperature-controlled cleavage plasmid pUC-ΔWK-E was constructed. The MOMP and green fluorescent protein (GFP) genes were amplified using the primers (MOMP-F1/MOMP-R1) and (GFP-F1/GFP-R), respectively, and cloned into multiple cloning site 1 (MCS1) of pACYCDuet-1 and designated as pACYC-MOMP and pACYC-GFP, respectively. Primers (GFP-F2/GFP-R) were used to amplify the GFP gene and clone it into the MCS1 of pACYCDuet-1, and subsequently, the amplified E’ gene (E’-F1/E’-R) was cloned into the 5′ end of the GFP gene, resulting in the E’ and GFP gene sequences being fused. This plasmid was designated pACYC-E’-GFP. Similarly, primers (MOMP-F2/MOMP-R1) were used to amplify the MOMP gene and clone it into the MCS1 of pACYCDuet-1. Following this, the anchor sequences E’ (E’-F2/E’-R), InpN (InpN-F/InpN-R), MBP (MBP-F/MBP-R), and SbsA (SbsA-F/SbsA-R) were amplified and then cloned into the 5′ end of the MOMP gene, resulting in the fusion of the anchor and MOMP gene sequences. The constructed anchor plasmids were designated as pACYC-E’-MOMP, pACYC-InpN-MOMP, pACYC-MBP-MOMP, and pACYC-SbsA-MOMP. Additionally, the MOMP gene was amplified with primers (MOMP-F3 and MOMP-R2) and cloned into multiple cloning site 2 (MCS2) of pACYC-MOMP to construct pACYC-MOMP/MOMP.

### 2.4 Detection of the cleavage activity of recombinant temperature-controlled cleavage plasmids

The cleavage activity was determined as previously described (Yu et al., 2011). Briefly, *E. coli* transformed with different lysis plasmids was cultured at 30°C or 37°C until the OD_600_ was approximately 0.4, and subsequently the temperature was rapidly raised to 42°C to induce E protein expression. Following this, 100 μL of bacterial liquid was obtained before (0 h) and after induction (5 h) and spread onto LB solid medium containing 100 μg/mL kanamycin. The colonies were counted after culturing overnight. The following formula was used to calculate the bacterial lysis efficiency: lysis rate = (1- CFU _after_/CFU _before_) × 100%, and this was repeated three times for each sample. For bacteria co-transfected with antigen expression and pUC-ΔWK-E plasmids, antigen expression was first induced, and subsequently, the culture was diluted with fresh culture medium until reaching an OD_600_ of approximately 0.4. Following this, the temperature was increased to induce E protein expression. Colonies were grown and counted in LB solid medium containing 100 μg/mL kanamycin and 50 μg/mL chloramphenicol.

### 2.5 Confocal microscopy and transmission electron microscopy

Pre- and post-lysis *E. coli* F107/86 cells were fixed using 2.5% glutaraldehyde as described previously (Soleymani et al., 2020). The cell membranes were stained with sulforhodamine B (Merck, Germany), and nucleic acids were stained with Gel Green dye (Solarbio, China). For *E. coli* cells expressing the GFP protein, only sulforhodamine B was used to stain the membranes. Appropriate amounts of fixed bacteria were applied to a confocal dish and observed under a confocal microscope (Leica TCS SP8, Germany) after drying. The excitation wavelengths of sulforhodamine B, 4′,6-diamidino-2-phenylindole (DAPI), and Gel Green and GFP are 594 nm, 450 nm, and 488 nm, respectively.

TEM analysis was performed as previously described (Hu et al., 2019). Briefly, *E. coli* was fixed with a 2.5% glutaraldehyde solution for 2 h, dehydrated with an alcohol gradient, impregnated with resin to infiltrate the embedding agent, and polymerized into a hard solid. Finally, the ultrathin sections were analyzed using a transmission electron microscope (Hitachi HT7800, Japan) after sectioning with an ultramicrotome (Leica EM UC7, Germany). All images were exported to the ImageJ (version 1.8) software for analysis.

### 2.6 Flow cytometry

**Figure 1:**
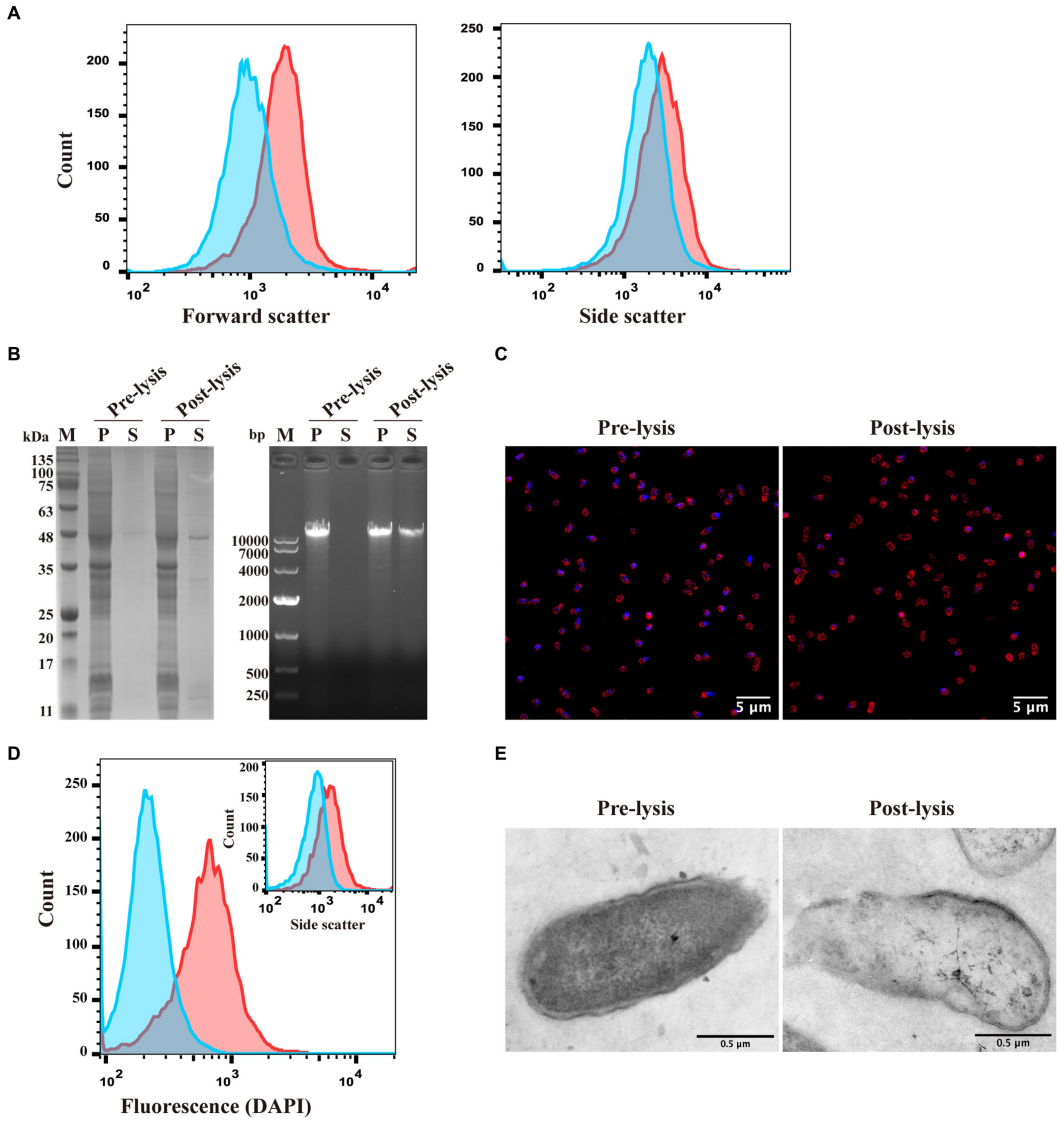
Construction of the recombinant temperature-controlled lysis plasmid pUC-ΔWK-E and its lysis curve in *E. coli*. **(A)** Schematic illustration of the construction of pUC-ΔWK-E. The detailed construction process is described in the “Construction of plasmids” section in the Materials and Methods. **(B)** Schematic representation of the operation/promoter sequence point mutations in the λP_R_ and P_RM_ promoters of the temperature-controlled system. The T to C base exchange in the O_R_2 region of the 41C promoter and the **A** to **G** base exchange at the P_R_ position of the 32G promoter are indicated by arrows. **(C,D)** Lysis curves of *E. coli* containing different lysis plasmids cultured at 30°C **(C)** or 37°C **(D)** before inducing E gene expression. Error bars represent the standard deviation (SD); *n* = 3.

**Figure 2:**
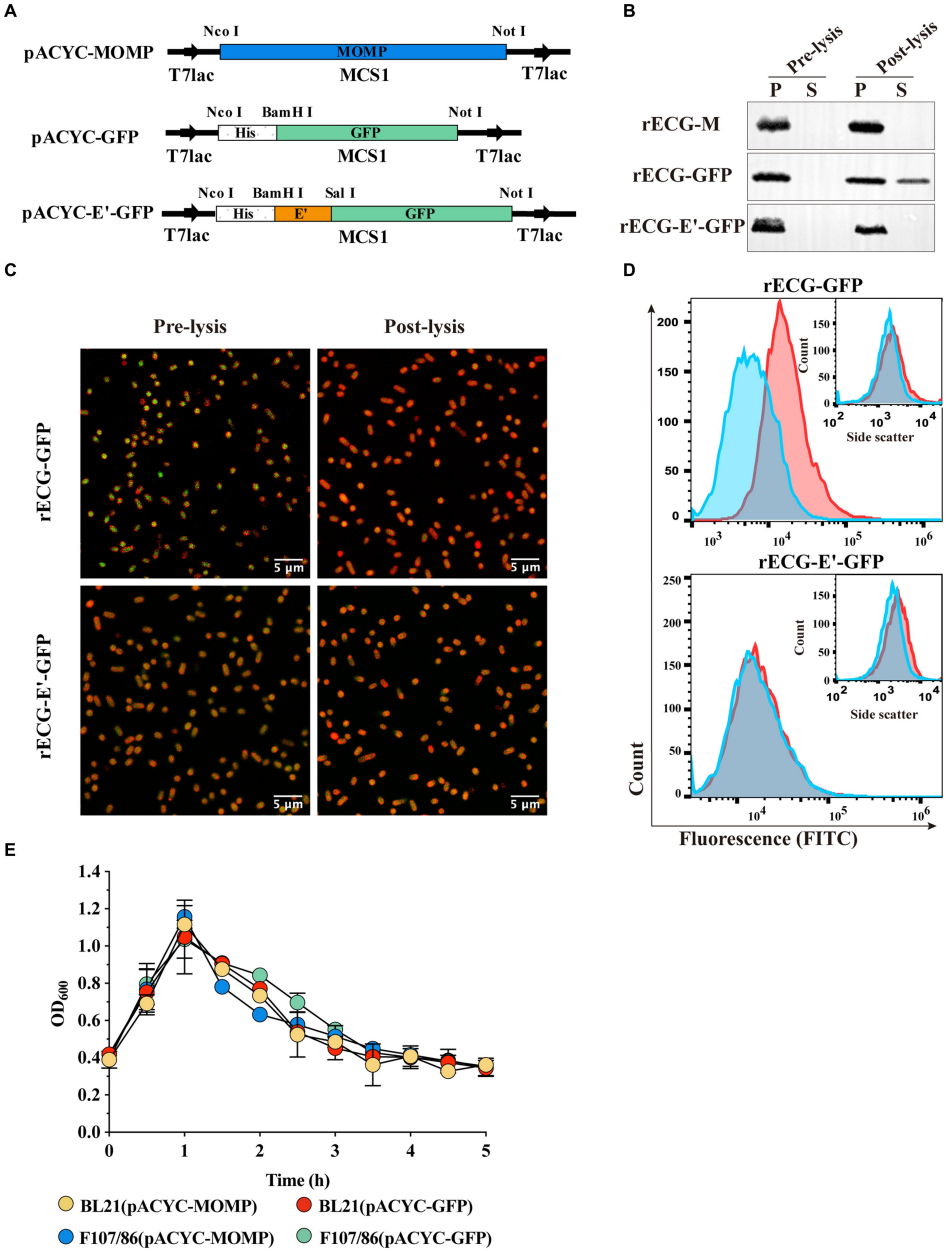
Characterization of *E. coli* ghosts formed by inducing lytic protein E expression using the pUC-ΔWK-E plasmid. **(A)** The sizes (forward scatter) and granularity (side scatter) of *E. coli* F108/86 pre-lysis and post-lysis were monitored using flow cytometry. Red line, pre-lysis; blue line, post-lysis. **(B)** Bacteria were analyzed for intracellular protein and genome leakage using SDS-PAGE (left panel) and agarose gel electrophoresis (right panel). M, Marker; P, pellet; S, supernatant. **(C)** Genome leakage in bacteria was observed using confocal microscopy. Bacterial cell membranes were labeled with sulforhodamine B (red), and the bacterial genome was labeled with DAPI (blue). Scalebars = 5 μm. **(D)** The fluorescence microscopy analysis of the bacteria shown in panel C was verified at the population level using flow cytometry. Both DAPI fluorescence and granularity (inset) were monitored. Red line, pre-lysis; blue line, post-lysis. **(E)** The pre-lysis and post-lysis bacteria in panel C were fixed, stained, and visualized using TEM. Scalebars = 0.5 μm.

Samples were prepared as described in the “Confocal Microscopy” section, and bacterial size (forward scatter), particle size (side scatter), and fluorescence (cytoplasmic GFP and nucleic acid dye DAPI) were analyzed using flow cytometry (Beckman CytoFLEX LX) as previously described (Hjelm et al., 2015). The cells were labeled with sulforhodamine B to exclude background interference. Data processing and analysis were performed using the FlowJo (version 10.8.1) software. The experiments were repeated at least three times, and representative examples are shown in Figures 1A,D, 2D.

### 2.7 Production of recombinant *Escherichia coli* ghost vaccines

The antigen expression plasmid derived from pACYCDuet-1 and the pUC-ΔWK-E plasmid were co-transformed into *E. coli* F107/86 to prepare the rECG vaccine. Briefly, the co-transformed F107/86 cell culture was expanded to 500 mL in an LB liquid medium containing kanamycin (100 μg/mL) and chloramphenicol (50 μg/mL). When the OD_600_ reached approximately 0.3, 0.5 mM isopropyl-beta-D-thiogalactopyranoside was added to induce antigen expression, followed by a 12-h culture at 18°C and 150 rpm/min. Subsequently, the induced bacterial solution was diluted with a fresh culture medium until reaching an OD_600_ of approximately 0.4. The temperature was rapidly increased to 42°C to induce protein E expression, and this was followed by a 5-h culture at 200 rpm/min. The culture was washed with phosphate buffer solution (PBS) and prepared into lyophilized powder, which was stored at 4°C. Following this, 5 mg of freeze-dried powder was resuspended in LB, spread onto an LB solid culture plate containing 100 μg/mL kanamycin and 50 μg/mL chloramphenicol double antibody, and this was cultured at 37°C overnight. Complete inactivation was indicated if no colonies grew. Each experiment was repeated three times.

### 2.8 Bacterial subcellular fractionation

The cytoplasm, periplasm, inner membrane, and outer membrane components of the rECG vaccine were separated as previously described and stored at −80°C (Maurer et al., 1997).

### 2.9 SDS-PAGE and immunoblotting

Bacterial pellets, culture supernatants, bacterial subcellular fractions, and protein samples were mixed with 5 × sodium dodecyl sulfate (SDS) sample buffer and separated by performing SDS-polyacrylamide gel electrophoresis (SDS-PAGE) using a 10% resolving SDS-PAGE gel. The protein bands were visualized by staining with Coomassie Brilliant Blue R-250. The electrophoresis gel was transferred to a polyvinylidene difluoride membrane for western blot analysis. The membrane was blocked with 5% skim milk overnight at 4°C, incubated with primary antibody mouse anti-MOMP antibody or mouse anti-His antibody for 1 h at 4°C, and then labeled with horseradish peroxidase (HRP) goat anti-mouse IgG antibody (Abcam; 1:5000) and incubated for 1 h at room temperature. Finally, the HRP chromogenic substrates were photographed after reacting.

### 2.10 Nucleic acid electrophoresis detection

DNA was extracted from bacteria and culture supernatant using a bacterial DNA extraction kit following the manufacturer’s instructions. The extracted DNA was mixed with 10 × loading buffer, and 20 μL was added per well for electrophoresis separation on a 2% agarose gel. Finally, photographic analysis was performed using a high-resolution image acquisition system (Chemdoc MP, USA).

### 2.11 Quantification of MOMP protein expression

Refer to Isoda et al. (2007) for quantifying the expression of MOMP using an enzyme-linked immunosorbent assay (ELISA). 96-well ELISA plates were coated with commercial monoclonal anti-MOMP IgG. Add purified recombinant MOMP protein gradient dilutions or bacterial lysate from the “SDS-PAGE” step to the plate and incubate. Apply biotinylated anti-MOMP IgG, then incubate with HRP-labeled avidin and then add 3,3′,5,5’-Tetramethylbenzidine (TMB) substrate for color development. Record the absorbance at 450 nm using a microplate reader. Draw a standard curve and calculate the protein content according to the standard curve equation. The total protein concentration of each lysate was determined using the bicinchoninic acid protein quantification kit (Solarbio, China). The specific MOMP concentration in each lysate was normalized to the total protein concentration to allow comparison of expression levels for each strain in terms of μg MOMP/mg total protein in the lysate.

### 2.12 Immunization, challenge, and analysis of protective immunity

Ninety six mice were randomly divided into eight groups (*n* = 12): subunit vaccine group, wherein each mouse was intramuscularly injected with recombinant MOMP antigen (rMOMP) 20 μg; empty vector group, wherein each mouse was injected with 2 mg *E. coli* ghost (ECG); and six recombinant *E. coli* ghost (rECG) vaccine groups: rECG-E’-M, rECG-InpN-M, rECG-MBP-M, rECG-SbsA-M, rECG-M, and rECG-M/M. Each group received a 2 mg intramuscular injection per mouse. Before immunization, 20 μg of rMOMP was emulsified with 100 μL of complete Freund’s adjuvant (Sigma, USA) for the initial immunization and with incomplete Freund’s adjuvant for the second and third immunizations. For each immunization, 2 mg of lyophilized bacterial shadow vaccine powder was resuspended in 100 μL of normal saline, with a total of three immunizations administered and a two-week interval between immunizations. Serum samples and vaginal douches were obtained two weeks after the second and last immunizations and stored at −80°C until analysis.

Two weeks after the last immunization, each mouse was intraperitoneally injected with medroxyprogesterone (2.5 mg) to synchronize estrous cycles and promote productive infection. Three weeks after the last immunization, 5 × 10^5^ inclusion-forming units (IFU) of *C. abortus* GN6 was inoculated vaginally to evaluate the protective effect. The infection level was assessed as described previously (Eko et al., 2011a). Briefly, a vaginal swab was collected every three days after the challenge, and *C. abortus* in the vaginal swab was isolated and cultured. *C. abortus* IFUs from the vaginal swab were counted using indirect immunofluorescence, and the average IFU number at each time point was calculated. Each experiment was performed in triplicate.

### 2.13 ELISA analysis for antibody levels

MOMP antigen-specific antibody levels in mouse serum and vaginal lavage fluid were measured using standard ELISA procedures as described previously (Macmillan et al., 2007) with appropriate modifications. Briefly, 96-well microtiter plates (Corning, NY) were coated with rMOMP protein (2.5 μg/well), diluted in PBS overnight at 4°C, and incubated with 1% bovine serum albumin (BSA). Vaginal wash solution or 50-fold diluted serum was added and incubated at room temperature. After incubation with HRP-labeled goat anti-mouse IgG, IgG2a, and IgA antibodies (Abcam, UK), TMB substrate was added to protect from light exposure and induce color development. Finally, H_2_SO_4_ was added to terminate the reaction, and the absorbance at 450 nm was measured using a microplate reader (TECAN, infinite M200 PRO). Results were generated simultaneously with the standard curve, and data sets representing the average of triplicate wells are shown as the mean concentration (ng/mL) ± SD. To detect *E. coli*-specific antibodies, inactivated *E. coli* cells were used for antigen coating, and the subsequent steps were similar to those for the MOMP antibody detection method mentioned above.

### 2.14 Lymphocyte proliferation assay

The mice were sacrificed one week after the last immunization, and spleen lymphocytes were collected to prepare a single-cell suspension. The suspension was added to a 96-well plate at 90 μL/well (10^6^ cells/mL), along with 10 μL of *C. abortus* antigen (10 μg/mL). After co-incubation for 72 h, 20 μL/well of Cell Counting Kit-8 (CCK-8) (Promega, China) was added and incubated for 4 h. Absorbance was measured at 450 nm using a microplate reader. The results are expressed as the stimulation index (SI), calculated using the following formula: SI = (stimulated cells OD_450_ – culture medium OD_450_)/(non-stimulated cells OD_450_ – culture medium OD_450_). Each sample was analyzed in triplicate.

### 2.15 Detection of cytokine production using ELISA

After incubating the spleen lymphocytes with the antigen for 72 h, the supernatant was collected, and the cytokines in the supernatant were detected following the operating procedures of the ELISA kit (R&D Systems, USA). Absorbance was measured at 450 nm using a microplate reader. A standard curve was constructed based on the OD_450_ values of the standard, and the cytokine content in each sample was calculated based on the standard curves of IFN-γ, IL-12, IL-4, and IL-10. The mean and SD of five replicate cultures were calculated.

### 2.16 Statistical analysis

Statistical analyses were performed using the GraphPad Prism 10 software. The statistical significance of the difference between two groups was evaluated using a Student *t*-test, and the differences between more than two groups were assessed using a one-way analysis of variance. Differences were considered statistically significant at *p < 0.05, **p < 0.01, or ***p < 0.001.

## 3 Results

### 3.1 Construction of the recombinant temperature-controlled lysis plasmid pUC-ΔWK-E and detection of cleavage activity

Figure 1A shows an illustration of the construction of the recombinant temperature-controlled lysis plasmid pUC-ΔWK-E. Referring to a previous study (Jechlinger et al., 2005a), the “WK-E” lysing cassette promoter was point-mutated (Figure 1B). The most visual method to assess the lytic activity of E protein was to measure the extent to which the OD_600_ value decreased (Ma et al., 2022). Therefore, the lysis activity of the pUC-ΔWK-E plasmid was evaluated in *E. coli* BL21 and pathogenic *E. coli* F107/86. The bacteria were cultivated at 30°C before inducing lysis, and when the temperature rose to 42°C, the OD_600_ of BL21 cells containing three different lysis plasmids gradually increased, reaching a peak (of approximately 1.0) at 1 h, and then decreased. After 3 h, the OD_600_ remained unchanged (Figure 1C). Colony forming units (CFU) counting showed that the lysis efficiency was >99.9% (Table 1). The OD_600_ of *E. coli* (BL21 and F107/86) containing the pUC-ΔWK-E plasmid cultured at 37°C before induced lysis also increased after the temperature was increased to 42°C and then gradually decreased to a stable level, and the lysis efficiency reached above 99.9% (Table 1). However, the OD_600_ of BL21 cells containing the pBV220-E and pUC-WK-E plasmids increased slowly, reaching a maximum value (of approximately 0.5) at 2 h, and then remained unchanged, with lysis efficiencies of 65.921 and 62.914%, respectively (Figure 1D; Table 1). The results show that the recombinant temperature-controlled lysis plasmid pUC-ΔWK-E was successfully constructed. This plasmid expanded the temperature range of bacterial culture; essentially, it can stably inhibit E gene expression at 37°C and exhibits high lytic activity in both engineered and pathogenic *E. coli*.

**Figure 3:**
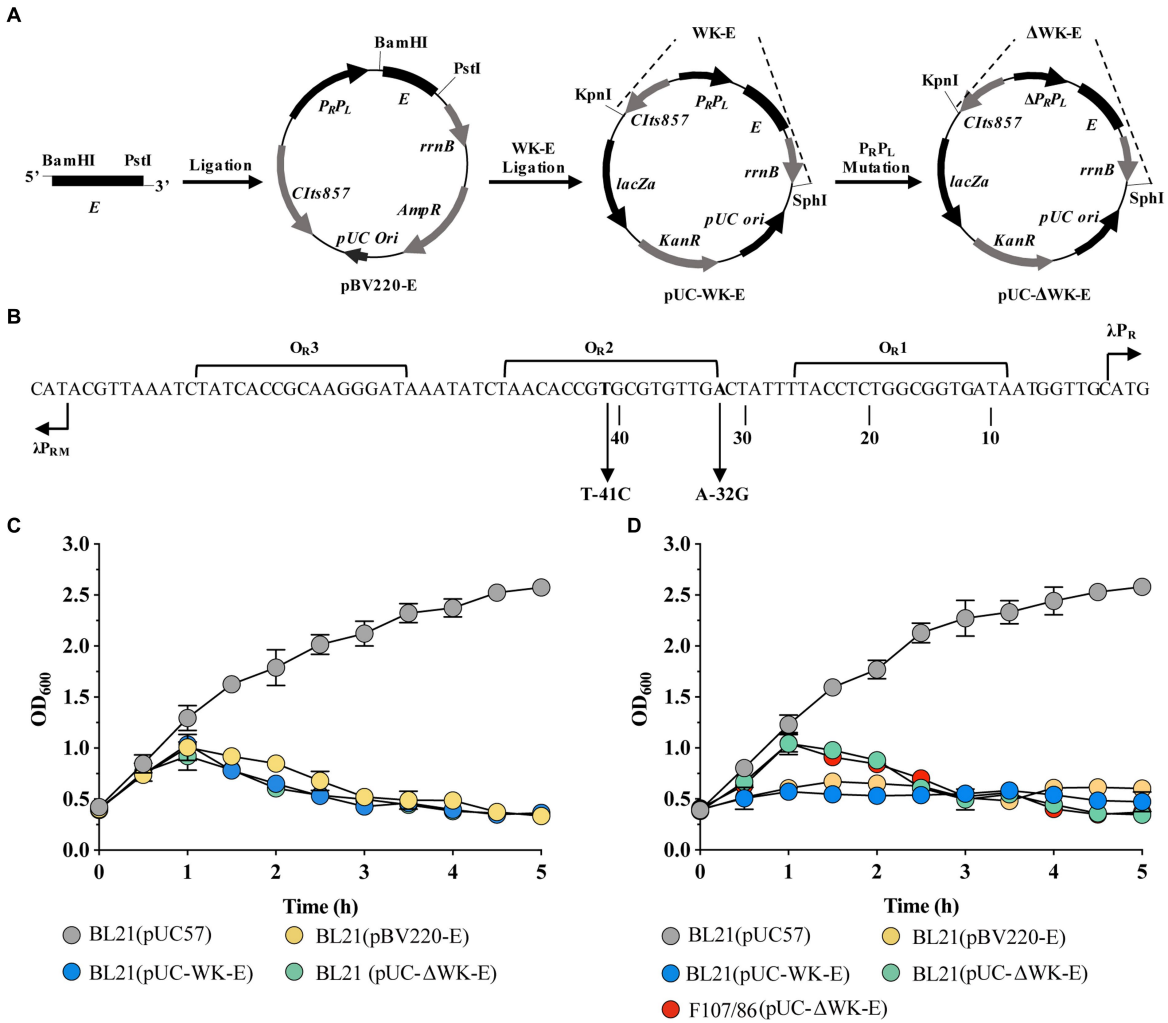
Expression and characterization of heterologous antigens in *E. coli* ghosts. **(A)** Schematic diagram of expression plasmid construction. MOMP and GFP were cloned into MCS1 on the pACYCDuet-1 vector, while the anchoring sequence E’ was fused to GFP and cloned into MCS1. **(B)** Western blot detected exogenous antigen leakage from *E. coli* F108/86 pre-lysis and post-lysis. P, pellet; S, supernatant. **(C)** Confocal observation of intracellular GFP (green). Bacterial cell membranes were labeled with sulforhodamine B (red). Scalebars = 5 μm. **(D)** GFP (FITC) fluorescence and granularity (inset) were monitored using flow cytometry. Red line, pre-lysis; blue line, post-lysis. **(E)** After inducing heterologous antigen expression, the OD_600_ of the bacterial solution was adjusted to approximately 0.4. Following this, the culture temperature was rapidly increased to 42°C to induce lytic protein E expression, and the OD_600_ value of the culture was measured every 0.5 h until it was constant. Error bars indicate the SD; *n* = 3.

**Table 1:** Comparison of cleavage efficiency of different lysis plasmids.

	Bacteria (Plasmid)	0 h (CFU)	5 h (CFU)	Bacterial Lysis rate (%)
30°C	BL21 (pBV220-E)	2.008 × 10^7^	1.026 × 10^4^	99.949
	BL21 (pUC-WK-E)	2.016 × 10^7^	1.493 × 10^4^	99.926
	BL21 (pUC-ΔWK-E)	1.982 × 10^7^	1.248 × 10^4^	99.937
37°C	BL21 (pBV220-E)	1.52 × 10^3^	5.18 × 10^2^	65.921
	BL21 (pUC-WK-E)	1.359 × 10^3^	5.04 × 10^2^	62.914
	BL21 (pUC-ΔWK-E)	2.217 × 10^7^	1.409 × 10^4^	99.936
	F107/86 (pUC-ΔWK-E)	1.996 × 10^7^	1.063 × 10^4^	99.947
37°C	BL21 (pUC-ΔWK-E + pACYC-MOMP)	2.243 × 10^7^	1.524 × 10^4^	99.932
	BL21 (pUC-ΔWK-E + pACYC-GFP)	2.194 × 10^7^	0.987 × 10^4^	99.955
	F107/86 (pUC-ΔWK-E + pACYC-MOMP)	2.361 × 10^7^	1.89 × 10^4^	99.92
	F107/86 (pUC-ΔWK-E + pACYC-GFP)	2.078 × 10^7^	1.978 × 10^4^	99.905

The data were presented in the format of mean values.

### 3.2 Characterization of *Escherichia coli* ghosts

To characterize the morphological changes and release of cytoplasmic content in *E. coli* before and after lysis, flow cytometry was used to detect the forward scatter (FSC) and side scatter (SSC) of the bacteria before and after E protein expression. These indicators represented bacterial size and internal complexity, respectively (Hjelm et al., 2015). The size of the bacteria after lysis was smaller than that before lysis. Moreover, the granularity size was significantly lower than that before lysis, indicating that at least part of the cytoplasmic content was lost (Figure 2A). Subsequently, we analyzed the presence of proteins and genomes in the cytoplasm before and after lysis. SDS-PAGE and nucleic acid electrophoresis experiments revealed that after E protein expression was induced, part of the cytoplasmic protein and genome were released into the extracellular environment (Figure 2B).

Subsequently, confocal microscopy and TEM were used to observe the morphology of the *E. coli* cells. After E protein expression was induced, the cytoplasmic nucleic acid signal (blue) diminished. However, cell membranes (red) remained visible (Figure 2C). Flow cytometry performed on the same samples revealed similar results to the confocal microscopy, wherein the nucleic acid fluorescence signal of the lysed bacteria was attenuated or disappeared (Figure 2D). In the TEM images, the ghosts had a more transparent appearance than the non-lysed bacteria owing to the reduced electron density, while the cell membranes were still retained. The contours of the cell membrane-formed ghosts were not significantly different from those of control bacteria (Figure 2E).

### 3.3 The impact of the antigen expression form on the leakage of heterologous antigens expressed by ECGs

To investigate whether expressed heterologous antigens leak during BG formation and whether leakage is related to antigen expression form, we constructed plasmids expressing the *C. abortus* MOMP protein and GFP (Figure 3A). The MOMP protein was expressed in inclusion bodies, whereas GFP was expressed in a soluble form (Supplementary Figure 1). These two plasmids were co-transfected with pUC-ΔWK-E into *E. coli* F107/86, respectively, to induce exogenous protein expression before warming to 42°C to induce E protein expression. The western blot results showed that for the rECG-M vaccine, MOMP protein was only detected in the precipitate before and after lysis. Similarly, in the rECG-GFP group, GFP protein was also detected in the culture supernatant after lysis. When the GFP protein was fused and expressed with the anchoring sequence E’ (Figure 3A), it was undetectable in the supernatant (Figure 3B). We also observed a similar phenomenon using the *Mycobacterium tuberculosis* Hsp70 protein (soluble form) (Supplementary Figures1, 2). Leakage of the GFP protein was further detected using laser confocal microscopy and flow cytometry. Compared with before lysis, most of the GFP protein in rECG-GFP leaked after lysis, resulting in weakened green fluorescence, while the green fluorescence signal intensity of rECG-E’-GFP remained essentially unchanged (Figure 3C). Flow cytometry also verified the results of the confocal microscopy (Figure 3D). The above results indicate that the leakage of heterologous antigens expressed by ECGs is related to the expression form of the antigen. Additionally, the anchor sequence can anchor the soluble protein onto the bacteria so that it will not leak during ghost formation.

In addition, we examined the cleavage activity of the E protein after the expression of exogenous antigens. The trend observed in the cleavage curve when E protein expression was induced after the exogenous antigen was expressed in *E. coli* (BL21 and F107/86) was similar to that observed in the lysis curve when only E protein expression was induced (Figure 3E). The CFU counts showed that the cleavage efficiencies were all above 99.9% (Table 1). This indicated that exogenous antigen expression did not affect the cleavage efficiency of the E protein.

### 3.4 Construction and characterization of a recombinant *Escherichia coli* ghost vaccine expressing the MOMP antigen

**Figure 4:**
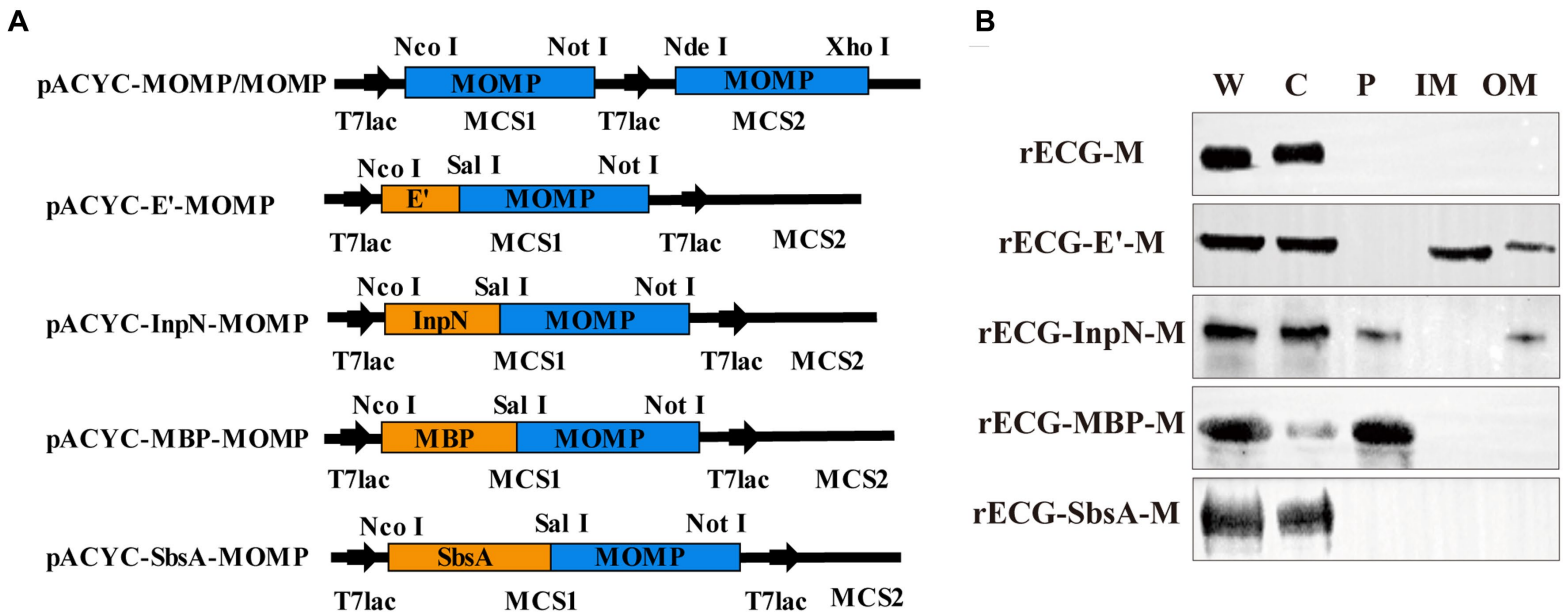
Preparation and characterization of the recombinant *E. coli* ghost vaccine. **(A)** Schematic representation of the plasmid construct expressing the MOMP antigen. The detailed construction process is described in the “Construction of plasmids” section in the Materials and Methods. **(B)** Western blot to detect the expression of MOMP fusion proteins at different locations in the rECGs. W, whole cells; C, cytoplasmic fractions; P, periplasmic space; membrane fractions; OM, outer membrane fractions.

Four anchoring sequences were cloned into the N-terminus of the MOMP gene sequence, and fusion expression plasmids of the anchoring sequences and MOMP gene were constructed. In addition, a pACYC-MOMP/MOMP plasmid co-expressing the two MOMP proteins was constructed (Figure 4A). They were then co-transformed with pUC-ΔWK-E into *E. coli* F107/86 to prepare rECG vaccines. To determine whether the MOMP proteins were anchored to the appropriate positions on the rECGs, the components of the rECG vaccine were separated using western blotting. The bands of the fusion proteins were detected at the corresponding positions of the four rECG vaccines. Furthermore, we found that bands for rECG-E’-M, rECG-InpN-M, and rECG-MBP-M were detected in the cytoplasm, while the rECG-InpN-M bands were detected in the periplasmic space (Figure 4B).

Furthermore, we performed ELISA to quantify MOMP antigen expression in the rECG vaccine (Table 2). The antigen content was calculated based on the OD_450_ value of the reaction solution using a known concentration of recombinant MOMP protein as a reference. The MOMP antigen content in different vaccines varied considerably. The MOMP protein content in rECG-M/M peaked at 8.9 μg/mg, which was 1.7 times higher than that in rECG-M. The lowest MOMP content was observed in the rECG-SbsA-M vaccine at 0.88 μg/mg. In addition, live bacteria were not detected in the prepared rECG vaccines, indicating that all the vaccines were fully inactivated.

**Table 2:** MOMP expression levels in *E. coil* strains.

Vaccine	MOMP (μg/mL)	Total protein (μg/mL)	MOMP/Total protein (μg/mg)
rECG-E’-M	3.4	3,312	1.03
rECG-InpN-M	8.6	3,068	2.8
rECG-MBP-M	22.3	4,002	5.57
rECG-SbsA-M	2.6	2,947	0.88
rECG-M	18.2	3,464	5.25
rECG-M/M	31.4	3,527	8.9

The data were presented in the format of mean values.

### 3.5 The rECG vaccine induces strong humoral immune responses specific to MOMP antigens

**Figure 5:**
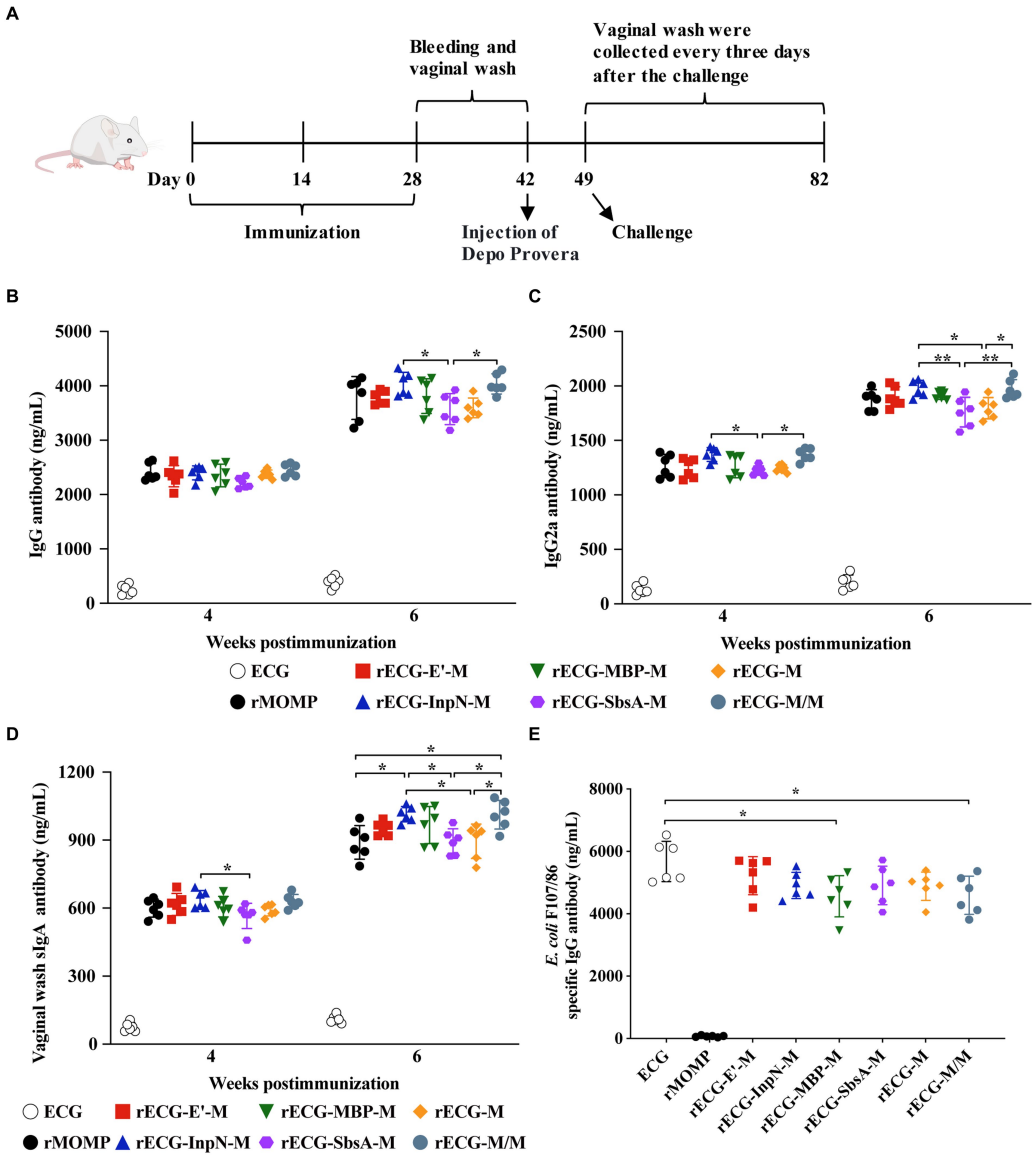
Detection of antibody levels in immunized mice. **(A)** Immunization scheme used for the protection assay. **(B–D)** IgG **(B)** and IgG2a **(C)** antibody levels in the serum and sIgA **(D)** antibody levels in the vaginal washes of mice with anti-MOMP at 4 and 6 weeks after the first immunization. **(E)** Levels of IgG antibodies against *E. coli* F107/86 in serum. Data were calculated as the mean values (± SD); *n* = 6. *p < 0.05; **p < 0.01.

BALB/c mice were immunized intramuscularly with the rECG vaccine (Figure 5A). There was no significant difference in serum anti-MOMP-specific IgG antibody levels among all vaccine groups four weeks after the first immunization. However, six weeks after the first immunization, the serum IgG antibody levels in the rECG-InpN-M and rECG-M/M groups were significantly higher than those in the rECG-SbsA-M group (Figure 5B). Four weeks after the first immunization, serum IgG2a levels in the rECG-InpN-M and rECG-M/M groups were significantly higher than those in the rECG-SbsA-M group. By week 6, serum IgG2a levels in these two groups were significantly higher than those in the rECG-M and rECG-SbsA-M groups (Figure 5C). Additionally, the soluble IgA (sIgA) antibody content in the vaginal washes of immunized mice was determined. Four weeks after the first immunization, the sIgA content in the rECG-InpN-M group was higher than that in the rECG-SbsA-M group. By week 6, the serum sIgA levels in the rECG-InpN-M and rECG-M/M groups were significantly higher than those in the recombinant MOMP (rMOMP), rECG-SbsA-M, and rECG-M groups (Figure 5D). In addition, we detected the levels of specific antibody against *E. coli* F107/86 in mouse serum. Except for the rMOMP group, all the other seven groups produced high levels of specific antibodies. The antibody levels in the ECG group were significantly higher than those in the rECG-MBP-M and rECG-M/M groups (Figure 5E). These data indicate that the rECG vaccine can induce a strong humoral immune response against the MOMP antigen. Bacterial ghost vectors can also induce high levels of antibody production.

### 3.6 The rECG vaccine induces significant proliferation and high-level IFN-γ secretion in mouse spleen lymphocytes

**Figure 6:**
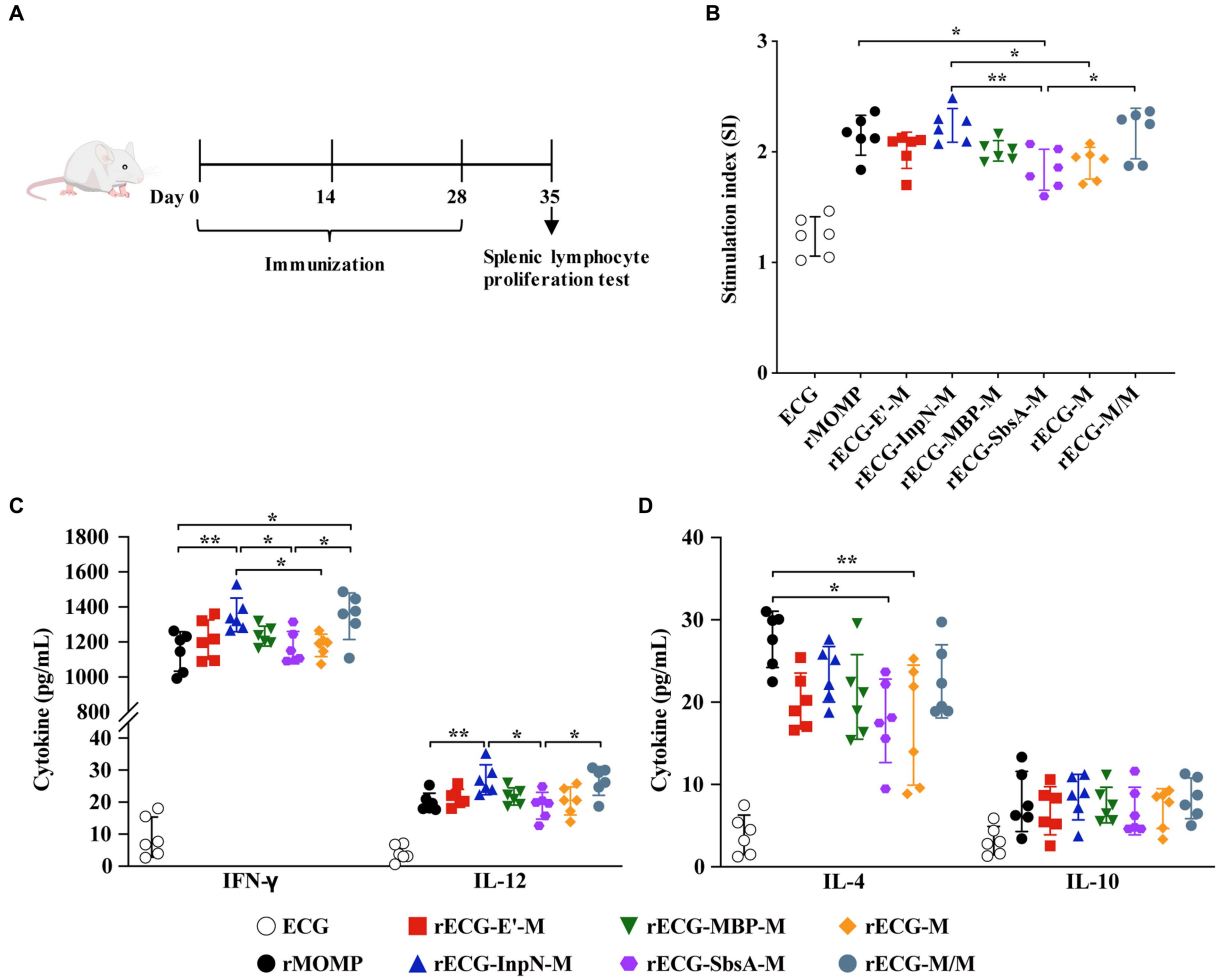
Cellular immune responses in immunized mice. **(A)** Scheme of the immunization regimen. **(B)** Specific splenic lymphocyte proliferative responses in immunized mice were detected using the CCK-8 reagent. **(C,D)** Levels of IFN-γ and IL-12 **(C)** and IL-4 and IL-10 **(D)** in the serum samples from immunized mice 1 week after the last immunization. Data were calculated as the mean values (± SD); *n* = 6. *p < 0.05; **p < 0.01.

The mouse model was used to further explore the exogenous antigen-specific cellular immune responses induced by the rECG vaccine. Splenic lymphocytes were collected 7 days after the last immunization. Proliferation and cytokine secretion were measured after re-stimulation with the rMOMP antigen (Figure 6A). The splenic lymphocyte proliferation response of mice immunized with rECG-InpN-M was significantly higher than that of mice in the rECG-SbsA-M and rECG-M groups. The rECG-M/M group also exhibited significantly higher levels than the rECG-SbsA-M group. Notably, the rMOMP group had significantly higher levels than the rECG-SbsA-M group (Figure 6B). The high IFN-γ levels induced by the rECG vaccine confirmed the results of the antibody response in Figure 5. The amount of the IFN-γ produced by spleen lymphocytes in mice immunized with rECG-InpN-M and rECG-M/M was significantly higher than that of mice immunized with rMOMP and rECG-SbsA-M. Moreover, the IFN-γ levels in the rECG-InpN-M group were significantly higher than those in the rECG-M group. Although the IL-12 secretion levels were much lower than those for IFN-γ, they exhibited a similar trend to IFN-γ. The rECG-InpN-M and rECG-M/M groups exhibited significantly higher levels than the rECG-SbsA-M group. Similarly, the levels in the rECG-InpN-M group were significantly higher than those of the rMOMP group (Figure 6C). Low levels of IL-4 and IL-10 were also observed in all vaccine groups. However, IL-4 levels in the rMOMP group were significantly higher than those in the rECG-SbsA-M and rECG-M groups (Figure 6D). The above results show that rECG induces mice to produce high levels of IFN-γ and low levels of IL-4 and IL-10.

### 3.7 MOMP antigen-loaded rECG vaccine significantly prevents *Chlamydia abortus* infection

**Figure 7:**
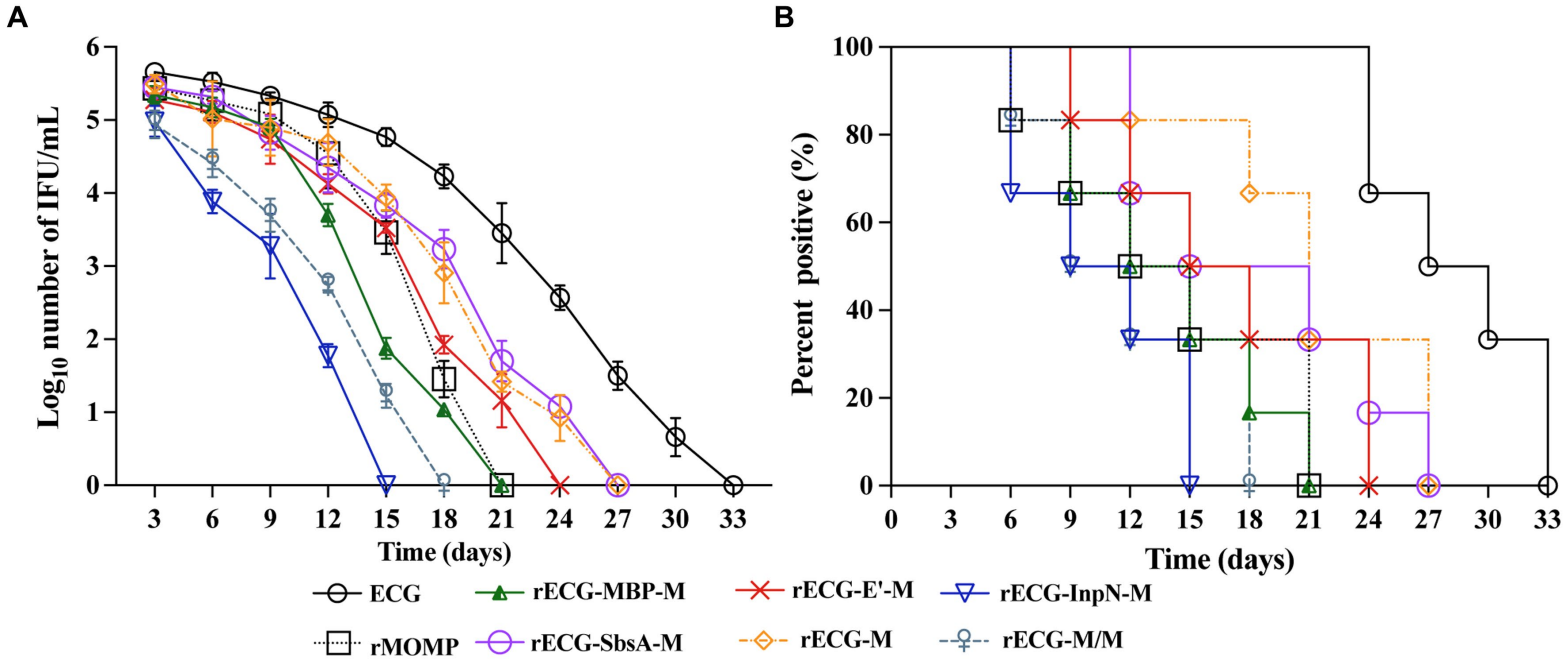
Protection against intravaginal challenge with the *C. abortus* GN6 strain. 3 weeks after the last immunization, immunized mice were challenged intravaginally with 5 × 10^5^ IFU of the *C. abortus* GN6 strain. Cervicovaginal swabs were collected every 3 days to monitor infection, and *Chlamydia* was isolated from swabs in tissue culture and quantified. **(A)** The mean recoverable IFUs are expressed as log_10_ IFU/mL ± SD. **(B)** The number (%) of mice with positive vaginal cultures at different times *n* = 6.

We determined whether the rECG vaccine could effectively reduce the shedding of *C. abortus*. 3 weeks after the last immunization, the reproductive tracts of the mice were challenged with 5 × 10^5^ IFU of the *C. abortus* GN6 strain and periodically monitored for the number of chlamydial IFUs shed (Figure 5A). From days 3 to 15 post-challenge, the rECG-InpN-M group had a significantly higher infection clearance rate than the other groups, clearing the infection on day 15. In contrast, the rMOMP group cleared the infection by day 21. In addition, the rECG-M/M group cleared the infection nine days earlier than the rECG-M group (Figure 7A).

We further evaluated the proportion of mice positive for *C. abortus* shedding in each group at each time point. The proportion of mice positive for *C. abortus* shedding at each time point was consistent with the trend observed for the number of *C. abortus* shedding cells. On day 15 after the challenge, no *C. abortus* shedding-positive mice were observed in the rECG-InpN-M group. On day 18, there were no shedding-positive mice in the rECG-M/M group, and in contrast, there were still 50% shedding-positive mice in the rECG-M group. *C. abortus* shedding was not detected in any mice in the ECG group on day 33 (Figure 7B). These results indicate that the rECG-InpN-M vaccine had the highest clearance efficiency for *C. abortus*, and the rECG-M/M vaccine co-expressing the MOMP antigen had a higher clearance efficiency for *C. abortus* than the rECG-M vaccine expressing MOMP alone.

## 4 Discussion

Given the insufficient protective efficacy of current commercially available inactivated vaccines and the risk of virulence returning with live attenuated vaccines, there is an urgent need for a safe and efficient vaccine to prevent *C. abortus* infections. The novel recombinant BG delivery platform is an efficient delivery system for chlamydial proteins that can induce significant immune protection without the addition of adjuvants (Ekong et al., 2009). However, for the widespread adoption of BG vectors for use in vaccine development, it is necessary to develop a more convenient and efficient BG preparation system and simultaneously explore different strategies to enhance the capacity of BG vaccines to induce more efficient immune responses. In this study, we controlled E gene expression using the temperature-inducible expression plasmid pBV220 without adding other chemicals (Ma et al., 2022). E gene expression is typically strictly suppressed at 28°C. At temperatures above 30°C, the repressor protein cI857 is inactivated, leading to the induction of E gene expression. Cultivation of bacteria below 30°C is not conducive to efficient BG production and the maintenance of critical antigenic determinants on the surface of some bacteria (Hu et al., 2019; Fu et al., 2021). This is not conducive to the efficient production of bacterial shadows. Therefore, to extend the temperature range of bacterial cultures, we introduced a point mutation in the pR/pL promoter (Jechlinger et al., 2005b). The mutated recombinant temperature-controlled cleavage plasmid could stably repress the expression of target proteins at 37°C and maintain extremely high cleavage efficiency. Moreover, the expression of exogenous antigens did not affect its cleavage activity. This establishes the foundation for the convenient and efficient production of ECG vaccines.

During the E protein-induced lysis of *E. coli* to form a ghost, the granularity (SSC) decreases owing to the outflow of cytoplasmic contents through the pores formed by lysis. The size of the bacteria (FSC) also decreased significantly, and this decrease in FSC may be due to the crumpling phenomenon in the bacteria due to external pressure (Hjelm et al., 2015), or it may be related to the change in the refractive index of the bacterial solution (Haidinger et al., 2003). However, the resulting ECGs maintain an intact outer membrane structure, which is fundamental for their utility as delivery carriers. Furthermore, we found that only when soluble proteins were fused to anchor sequences or expressed as inclusion bodies, they protected from leakage during E protein cleavage. Essentially, whether the foreign protein leaks when expressed in the BGs depends on the expression form of the antigen or whether it is fused with an anchor sequence. We analyzed that this may also be related to the size of the antigen, as the pores formed by the cleavage of the E protein have a specific size range (40 ~ 200 nm) (Jalava et al., 2003), and only proteins of an appropriate size can be released from the pores.

The role of Freund’s adjuvants is to enhance the immunostimulatory effect of antigens, especially in the case of weakly immunogenic subunit vaccines, and adding adjuvants can effectively enhance their immune effect. However, rECG vaccines have been shown to induce equal or even stronger immune responses than subunit vaccines without the addition of additional adjuvants, and they induce high levels of Th1-related IgG2a antibody and IFN-γ and genital tract-soluble IgA, which is also necessary for resistance to chlamydial infection (Eko et al., 2003). This suggests that the bacterial ghost carrier has a strong adjuvant effect, confirmed in other studies (Jiao et al., 2018). Exogenous antigens present in different parts of a delivery vehicle often elicit different immune responses (Hess et al., 1996; Muralinath et al., 2011; Hjelm et al., 2015). The immunological effects of several rECG vaccines we prepared also varied, with the use of the InpN sequence to display MOMP on the surface of ECGs had the best immune effect and could clear chlamydial infections first. This is consistent with the results of studies wherein antigens were displayed on bacterial surfaces and outer vesicles (Bermúdez-Humarán et al., 2004; Kajikawa et al., 2011; Kuipers et al., 2015), indicating that the immune effects of ECG vaccines are closely linked to the location of antigen presentation. Antigens exposed outside the bacteria can interact directly with immune cells and may be processed by antigen-presenting cells more efficiently than antigens encapsulated inside the bacteria (Kajikawa et al., 2011). Although the E’ sequence is currently the most commonly used anchor sequence (Eko et al., 2004, 2011b), in this study, we found that the fusion of E’ with MOMP is not conducive to protein expression. Moreover, the rECG-E’-M vaccine contained only 1.03 μg/mg of the target antigen (Table 2). The reason for this could be related to the transmembrane structure contained in the E’ sequence. Typically, the transmembrane structure of a protein is not conducive to its expression in prokaryotic expression systems (Cunningham and Deber, 2007). Similarly, rECG-SbsA-M had the lowest antigen content among all vaccines. The size of the SbsA protein can be as high as approximately 169 kDa. Large-molecular-weight proteins are also difficult to express in prokaryotic expression systems, decreasing the quantity of the target antigen. Therefore, these two vaccines have poor immune efficacy compared with rECG-MBP-M and rECG-InpN-M. This indicates that the immune effect may also be related to the quantity of the antigen (Riedmann et al., 2003). This was confirmed by comparing the rECG-M and rECG-M/M vaccines. Additionally, the immune effects of surface-displayed antigens may be related to immune pathways. When administered orally, gastric acid can easily destroy the antigens, resulting in a loss of activity (Reveneau et al., 2002; Perez et al., 2005). Therefore, the immune effect of the rECG vaccine is closely related to the antigen display site, amount, and immune pathway.

Interestingly, we found that when expression is induced at low temperatures (18°C), the InpN sequence only displays a portion of the MOMP on the ECG surface, while most of the antigen remains in the cytoplasm or periplasmic space. A similar phenomenon was observed with the MBP-anchored MOMP vaccine (rECG-MBP-M). This may be because of the use of an antigen expression system with a strong promoter, which results in the expression rate of the antigen being considerably higher than the anchoring rate, thereby resulting in it accumulating in the cytoplasm (Mathieu et al., 2019). We presume that by attempting to use a low-temperature promoter or optimizing the induction conditions to slow down the rate of antigen expression to maximize antigen presentation on the ECG surface, the immune effects may be further improved. Additionally, the corresponding bands for the rECG-E’-M vaccine were not only detected in the inner membrane, but a faint band was also visible in the outer membrane (Figure 4B). The possible reason for this phenomenon is that phage protein E cleavage induces the fusion of the bacterial inner and outer membranes, and the E’ sequence anchors the MOMP protein to the fusion site in the inner and outer membranes, enabling the detection of the target protein in the outer membrane (Jechlinger et al., 2005b).

Consistent with the results of this study, immunizing mice with the *C. abortus* vaccine using *Vibrio cholerae* ghosts as carriers also induced high antigen-specific IFN-γ, sIgA, and IgG2a antibody levels and enhanced protective immunity (Pan et al., 2015). The limitations of this study are that neither this nor previous studies have conducted infection protection tests in pregnant mouse models or large animals (pigs, sheep, and cattle), which will be the focus of our future research. In summary, we demonstrated that the *C. abortus* MOMP subunit vaccine vectored with pathogenic *E. coli* exhibited excellent immune efficacy. This adjuvant-free vaccine had more protective efficacy than the rMOMP subunit vaccine emulsified with Freund’s adjuvant, and the immune effect was best when the MOMP antigen was anchored to the ECG surface. This vaccine delivery vehicle, which does not require additional adjuvants, is easy to produce, inexpensive, and does not require a cold chain, has immeasurable application value.

## Data availability statement

The original contributions presented in the study are included in the article/Supplementary material, further inquiries can be directed to the corresponding authors.

## Ethics statement

The animal study was approved by Animal Ethics Committee of the Lanzhou Veterinary Research Institute, Chinese Academy of Agricultural Sciences. The study was conducted in accordance with the local legislation and institutional requirements.

## Author contributions

HZ: Conceptualization, Methodology, Data curation, Writing – review & editing. ZL: Conceptualization, Writing – review & editing, Funding acquisition, Resources. WL: Software, Supervision, Writing – review & editing. YJ: Investigation, Methodology, Writing – review & editing. YL: Formal analysis, Visualization, Writing – review & editing. QX: Data curation, Methodology, Writing – review & editing. DT: Conceptualization, Project administration, Writing – review & editing. JZ: Conceptualization, Investigation, Methodology, Writing – original draft.

## References

[ref1] Bao S. Yu S. Guo X. Zhang F. Sun Y. Tan L.. (2015). Construction of a cell-surface display system based on the N-terminal domain of ice nucleation protein and its application in identification of mycoplasma adhesion proteins. J. Appl. Microbiol. 119, 236–244. doi: 10.1111/jam.12824 25857598

[ref2] Bermúdez-Humarán L. G. Cortes-Perez N. G. Le Loir Y. Alcocer-González J. M. Tamez-Guerra R. S. De Oca-Luna R. M.. (2004). An inducible surface presentation system improves cellular immunity against human papillomavirus type 16 E7 antigen in mice after nasal administration with recombinant lactococci. J. Med. Microbiol. 53, 427–433. doi: 10.1099/jmm.0.05472-0 15096553

[ref3] Chatzikleanthous D. Schmidt S. T. Buffi G. Paciello I. Cunliffe R. Carboni F.. (2020). Design of a novel vaccine nanotechnology-based delivery system comprising CpGODN-protein conjugate anchored to liposomes. J. Control. Release 323, 125–137. doi: 10.1016/j.jconrel.2020.04.001, PMID: 32247804

[ref4] Chen H. Ji H. Kong X. Lei P. Yang Q. Wu W.. (2021). Bacterial ghosts-based vaccine and drug delivery systems. Pharmaceutics 13:1892. doi: 10.3390/pharmaceutics13111892, PMID: 34834306 PMC8622331

[ref5] Cunningham F. Deber C. M. (2007). Optimizing synthesis and expression of transmembrane peptides and proteins. Methods 41, 370–380. doi: 10.1016/j.ymeth.2006.07.003, PMID: 17367709

[ref6] Ebensen T. Paukner S. Link C. Kudela P. De Domenico C. Lubitz W.. (2004). Bacterial ghosts are an efficient delivery system for DNA vaccines. J. Immunol. 172, 6858–6865. doi: 10.4049/jimmunol.172.11.6858, PMID: 15153504

[ref7] Eko F. O. Ekong E. He Q. Black C. M. Igietseme J. U. (2011a). Induction of immune memory by a multisubunit chlamydial vaccine. Vaccine 29, 1472–1480. doi: 10.1016/j.vaccine.2010.12.024 21184858 PMC3032637

[ref8] Eko F. O. He Q. Brown T. McMillan L. Ifere G. O. Ananaba G. A.. (2004). A novel recombinant multisubunit vaccine against *Chlamydia*. J. Immunol. 173, 3375–3382. doi: 10.4049/jimmunol.173.5.3375, PMID: 15322201

[ref9] Eko F. O. Lubitz W. McMillan L. Ramey K. Moore T. T. Ananaba G. A.. (2003). Recombinant *Vibrio cholerae* ghosts as a delivery vehicle for vaccinating against *Chlamydia trachomatis*. Vaccine 21, 1694–1703. doi: 10.1016/S0264-410X(02)00677-1, PMID: 12639492

[ref10] Eko F. O. Okenu D. N. Singh U. P. He Q. Black C. Igietseme J. U. (2011b). Evaluation of a broadly protective Chlamydia–cholera combination vaccine candidate. Vaccine 29, 3802–3810. doi: 10.1016/j.vaccine.2011.03.027 21421002 PMC3084325

[ref11] Ekong E. E. Okenu D. N. Mania-Pramanik J. He Q. Igietseme J. U. Ananaba G. A.. (2009). A *Vibrio cholerae* ghost-based subunit vaccine induces cross-protective chlamydial immunity that is enhanced by CTA2B, the nontoxic derivative of cholera toxin. FEMS Immunol. Med. Microbiol. 55, 280–291. doi: 10.1111/j.1574-695X.2008.00493.x, PMID: 19040663 PMC3062614

[ref12] Fu L. X. Gong J. S. Gao B. Ji D. J. Han X. G. Zeng L. B. (2021). Controlled expression of lysis gene E by a mutant of the promoter pL of the thermo-inducible λcI857-pL system. J. Appl. Microbiol. 130, 2008–2017. doi: 10.1111/jam.14690, PMID: 32358825

[ref13] Gong S. Nan N. Sun Y. He Z. Li J. Chen F.. (2020). Protective immunity elicited by VP1 chimeric antigens of bacterial ghosts against hand-foot-and-mouth disease virus. Vaccine 8:61. doi: 10.3390/vaccines8010061, PMID: 32024212 PMC7158693

[ref14] Haidinger W. Szostak M. P. Jechlinger W. Lubitz W. (2003). Online monitoring of *Escherichia coli* ghost production. Appl. Environ. Microbiol. 69, 468–474. doi: 10.1128/AEM.69.1.468-474.2003, PMID: 12514029 PMC152429

[ref15] Hajam I. A. Dar P. A. Won G. Lee J. H. (2017). Bacterial ghosts as adjuvants: mechanisms and potential. Vet. Res. 48:37. doi: 10.1186/s13567-017-0442-5 28645300 PMC5482964

[ref16] Hess J. Gentschev I. Miko D. Welzel M. Ladel C. Goebel W.. (1996). Superior efficacy of secreted over somatic antigen display in recombinant Salmonella vaccine induced protection against listeriosis. Proc. Natl. Acad. Sci. U. S. A. 93, 1458–1463. doi: 10.1073/pnas.93.4.1458, PMID: 8643654 PMC1079202

[ref17] Hjelm A. Söderström B. Vikström D. Jong W. S. P. Luirink J. De Gier J.-W. (2015). Autotransporter-based antigen display in bacterial ghosts. Appl. Environ. Microbiol. 81, 726–735. doi: 10.1128/AEM.02733-14, PMID: 25398861 PMC4277592

[ref18] Hu J. Zuo J. Chen Z. Fu L. Lv X. Hu S.. (2019). Use of a modified bacterial ghost lysis system for the construction of an inactivated avian pathogenic *Escherichia coli* vaccine candidate. Vet. Microbiol. 229, 48–58. doi: 10.1016/j.vetmic.2018.12.020, PMID: 30642598

[ref19] Isoda R. Simanski S. P. Pathangey L. Stone A. E. S. Brown T. A. (2007). Expression of a *Porphyromonas gingivalis* hemagglutinin on the surface of a Salmonella vaccine vector. Vaccine 25, 117–126. doi: 10.1016/j.vaccine.2006.06.085, PMID: 16942819

[ref20] Jalava K. Eko F. O. Riedmann E. Lubitz W. (2003). Bacterial ghosts as carrier and targeting systems for mucosal antigen delivery. Expert Rev. Vaccines 2, 45–51. doi: 10.1586/14760584.2.1.45 12901596

[ref21] Jechlinger W. Glocker J. Haidinger W. Matis A. Szostak M. P. Lubitz W. (2005a). Modulation of gene expression by promoter mutants of the lambdacI857/pRM/pR system. J. Biotechnol. 116, 11–20. doi: 10.1016/j.jbiotec.2004.10.002 15652426

[ref22] Jechlinger W. Haller C. Resch S. Hofmann A. Szostak M. P. Lubitz W. (2005b). Comparative immunogenicity of the hepatitis B virus core 149 antigen displayed on the inner and outer membrane of bacterial ghosts. Vaccine 23, 3609–3617. doi: 10.1016/j.vaccine.2004.11.078 15855021

[ref23] Jiao H. Yang H. Zhao D. Chen J. Zhang Q. Liang J.. (2018). Design and immune characterization of a novel *Neisseria gonorrhoeae* DNA vaccine using bacterial ghosts as vector and adjuvant. Vaccine 36, 4532–4539. doi: 10.1016/j.vaccine.2018.06.006, PMID: 29914847

[ref24] Kajikawa A. Nordone S. K. Zhang L. Stoeker L. L. LaVoy A. S. Klaenhammer T. R.. (2011). Dissimilar properties of two recombinant *Lactobacillus acidophilus* strains displaying Salmonella FliC with different anchoring motifs. Appl. Environ. Microbiol. 77, 6587–6596. doi: 10.1128/AEM.05153-11, PMID: 21784918 PMC3187123

[ref25] Kudela P. Koller V. J. Lubitz W. (2010). Bacterial ghosts (BGs)--advanced antigen and drug delivery system. Vaccine 28, 5760–5767. doi: 10.1016/j.vaccine.2010.06.087, PMID: 20619379

[ref26] Kuipers K. Daleke-Schermerhorn M. H. Jong W. S. P. Ten Hagen-Jongman C. M. Van Opzeeland F. Simonetti E.. (2015). Salmonella outer membrane vesicles displaying high densities of pneumococcal antigen at the surface offer protection against colonization. Vaccine 33, 2022–2029. doi: 10.1016/j.vaccine.2015.03.010, PMID: 25776921

[ref27] Langemann T. Koller V. J. Muhammad A. Kudela P. Mayr U. B. Lubitz W. (2010). The bacterial ghost platform system: production and applications. Bioeng. Bugs 1, 326–336. doi: 10.4161/bbug.1.5.12540 21326832 PMC3037582

[ref28] Li Z. Cao X. Fu B. Chao Y. Cai J. Zhou J. (2015). Identification and characterization of *Chlamydia abortus* isolates from yaks in Qinghai, China. Biomed. Res. Int. 2015:658519. doi: 10.1155/2015/658519, PMID: 26060818 PMC4427853

[ref29] Longbottom D. Fairley S. Chapman S. Psarrou E. Vretou E. Livingstone M. (2002). Serological diagnosis of ovine enzootic abortion by enzyme-linked immunosorbent assay with a recombinant protein fragment of the polymorphic outer membrane protein POMP90 of *Chlamydophila abortus*. J. Clin. Microbiol. 40, 4235–4243. doi: 10.1128/JCM.40.11.4235-4243.2002, PMID: 12409404 PMC139646

[ref30] Ma Y. Zhu W. Zhu G. Xu Y. Li S. Chen R.. (2022). Efficient robust yield method for preparing bacterial ghosts by *Escherichia coli* phage ID52 lysis protein E. Bioengineering 9:300. doi: 10.3390/bioengineering9070300, PMID: 35877351 PMC9311611

[ref31] Macmillan L. Ifere G. O. He Q. Igietseme J. U. Kellar K. L. Okenu D. M.. (2007). A recombinant multivalent combination vaccine protects against *Chlamydia* and genital herpes. FEMS Immunol. Med. Microbiol. 49, 46–55. doi: 10.1111/j.1574-695X.2006.00165.x, PMID: 17094789

[ref32] Mathieu K. Javed W. Vallet S. Lesterlin C. Candusso M.-P. Ding F.. (2019). Functionality of membrane proteins overexpressed and purified from *E. coli* is highly dependent upon the strain. Sci. Rep. 9:2654. doi: 10.1038/s41598-019-39382-0 30804404 PMC6390180

[ref33] Maurer J. Jose J. Meyer T. F. (1997). Autodisplay: one-component system for efficient surface display and release of soluble recombinant proteins from *Escherichia coli*. J. Bacteriol. 179, 794–804. doi: 10.1128/jb.179.3.794-804.1997, PMID: 9006035 PMC178762

[ref34] Mayr U. B. Walcher P. Azimpour C. Riedmann E. Haller C. Lubitz W. (2005). Bacterial ghosts as antigen delivery vehicles. Adv. Drug Deliv. Rev. 57, 1381–1391. doi: 10.1016/j.addr.2005.01.027, PMID: 15878634

[ref35] Muhammad A. Champeimont J. Mayr U. B. Lubitz W. Kudela P. (2012). Bacterial ghosts as carriers of protein subunit and DNA-encoded antigens for vaccine applications. Expert Rev. Vaccines 11, 97–116. doi: 10.1586/erv.11.149 22149712

[ref36] Muralinath M. Kuehn M. J. Roland K. L. Curtiss R. (2011). Immunization with *Salmonella enterica* Serovar typhimurium-derived outer membrane vesicles delivering the pneumococcal protein PspA confers protection against challenge with *Streptococcus pneumoniae*. Infect. Immun. 79, 887–894. doi: 10.1128/IAI.00950-10, PMID: 21115718 PMC3028854

[ref37] Norton P. M. Brown H. W. Wells J. M. Macpherson A. M. Wilson P. W. Le Page R. W. (1996). Factors affecting the immunogenicity of tetanus toxin fragment C expressed in *Lactococcus lactis*. FEMS Immunol. Med. Microbiol. 14, 167–177. doi: 10.1111/j.1574-695X.1996.tb00284.x, PMID: 8809553

[ref38] Onorini D. Donati M. Marti H. Biondi R. Levi A. Nufer L.. (2019). The influence of centrifugation and incubation temperatures on various veterinary and human chlamydial species. Vet. Microbiol. 233, 11–20. doi: 10.1016/j.vetmic.2019.04.012, PMID: 31176395

[ref39] Pan Q. Pais R. Ohandjo A. He C. He Q. Omosun Y.. (2015). Comparative evaluation of the protective efficacy of two formulations of a recombinant *Chlamydia abortus* subunit candidate vaccine in a mouse model. Vaccine 33, 1865–1872. doi: 10.1016/j.vaccine.2015.02.007, PMID: 25698486 PMC4380638

[ref40] Paukner S. Kohl G. Lubitz W. (2004). Bacterial ghosts as novel advanced drug delivery systems: antiproliferative activity of loaded doxorubicin in human Caco-2 cells. J. Control. Release 94, 63–74. doi: 10.1016/j.jconrel.2003.09.010, PMID: 14684272

[ref41] Paukner S. Stiedl T. Kudela P. Bizik J. Al Laham F. Lubitz W. (2006). Bacterial ghosts as a novel advanced targeting system for drug and DNA delivery. Expert Opin. Drug Deliv. 3, 11–22. doi: 10.1517/17425247.3.1.11 16370937

[ref42] Perez C. A. Eichwald C. Burrone O. Mendoza D. (2005). Rotavirus vp7 antigen produced by *Lactococcus lactis* induces neutralizing antibodies in mice. J. Appl. Microbiol. 99, 1158–1164. doi: 10.1111/j.1365-2672.2005.02709.x, PMID: 16238746

[ref43] Reveneau N. Geoffroy M.-C. Locht C. Chagnaud P. Mercenier A. (2002). Comparison of the immune responses induced by local immunizations with recombinant *Lactobacillus plantarum* producing tetanus toxin fragment C in different cellular locations. Vaccine 20, 1769–1777. doi: 10.1016/S0264-410X(02)00027-0, PMID: 11906764

[ref44] Riedmann E. M. Kyd J. M. Smith A. M. Gomez-Gallego S. Jalava K. Cripps A. W.. (2003). Construction of recombinant S-layer proteins (rSbsA) and their expression in bacterial ghosts – a delivery system for the nontypeable *Haemophilus influenzae* antigen *Omp26*. FEMS Immunol. Med. Microbiol. 37, 185–192. doi: 10.1016/S0928-8244(03)00070-1, PMID: 12832124

[ref45] Soleymani S. Tavassoli A. Hashemi Tabar G. Kalidari G. A. Dehghani H. (2020). Design, development, and evaluation of the efficacy of a nucleic acid-free version of a bacterial ghost candidate vaccine against avian pathogenic *E. coli* (APEC) O78:K80 serotype. Vet. Res. 51:144. doi: 10.1186/s13567-020-00867-w 33298146 PMC7724879

[ref46] Won G. Senevirathne A. Lee J. H. (2020). *Salmonella Enteritidis* ghost vaccine carrying the hemagglutinin globular head (HA1) domain from H1N1 virus protects against salmonellosis and influenza in chickens. Vaccine 38, 4387–4394. doi: 10.1016/j.vaccine.2020.04.077, PMID: 32402750

[ref47] Yu S. Peng W. Si W. Yin L. Liu S. Liu H.. (2011). Enhancement of bacteriolysis of shuffled phage PhiX174 gene E. Virol. J. 8:206. doi: 10.1186/1743-422X-8-206 21548934 PMC3115883

[ref48] Zhang H. Zhang Z. Li Y. Li W. Jin Y. Li Z.. (2023). Seroprevalence of *Chlamydia abortus* and *Brucella spp.* and risk factors for *Chlamydia abortus* in pigs from China. Acta Trop. 248:107050. doi: 10.1016/j.actatropica.2023.107050, PMID: 37875168

