# Supplementary material for: Comparison of the immune effects of the Chlamydia abortus MOMP antigen displayed in different parts of bacterial ghosts
Source: Front Microbiol. 2024 Feb 7;15:1349746. doi: 10.3389/fmicb.2024.1349746 (PMC10883653; doi:10.3389/fmicb.2024.1349746)
Supplement: Supplementary file 1 [file Data_Sheet_1.docx]

**
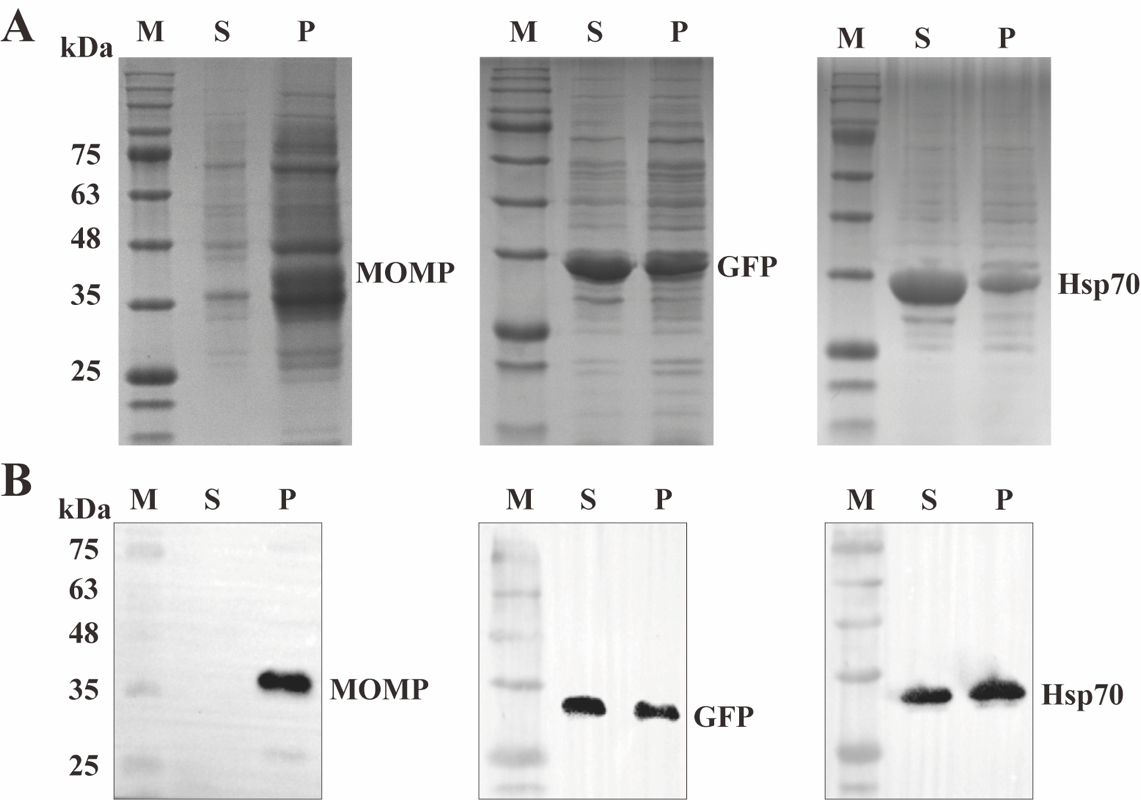
Supplementary Figure 1** Identification of protein expression forms. (A) SDS-PAGE and (B) western blot analysis of MOMP, GFP, and Hsp70 expression in *E. coli* F107/86. M, marker; S, supernatant after ultrasonic disruption; P, precipitation after ultrasonic disruption.

**
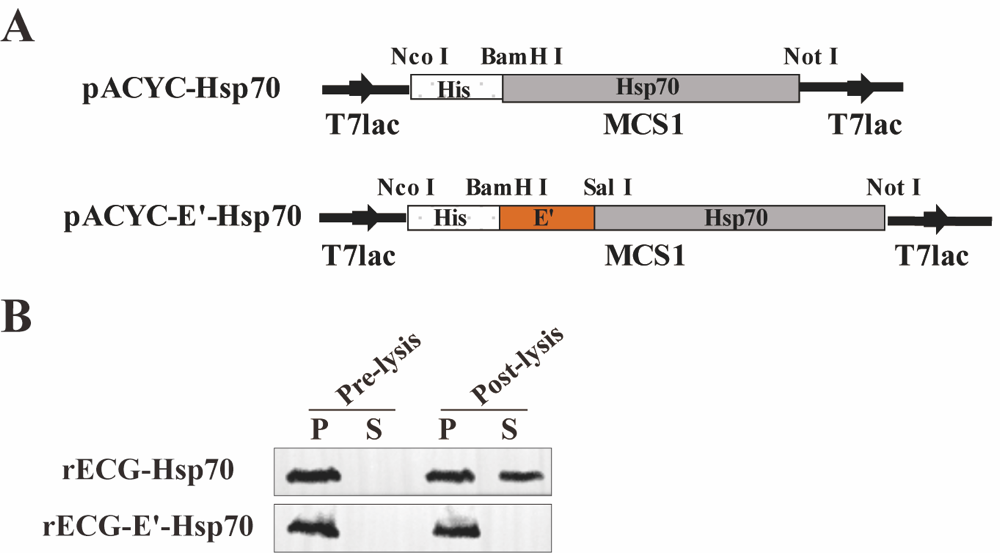
Supplementary Figure 2** (A) Schematic diagram of the construction of pACYC-Hsp70 and pACYC-E'-Hsp70 plasmids. (B) Western blot detecting leakage ofHsp70 protein from *E. coli* F108/86 pre-lysis and post-lysis. P, pellet; S, supernatant.

**Supplementary Table 1** Bacterial strains, plasmids, and cells used in this study.

| **Strain or plasmid** | **Characteristics** | **Reference or source** |
| --- | --- | --- |
| Bacterial strains |  |  |
| DH5α | cloning bacteria | Our lab |
| BL21(DE3) | expression bacteria | Our lab |
| *Escherichia coli* (F107/86) | porcine pathogenic *Escherichia coli*, serotype O139:H1 | Our lab |
| *Chlamydia abortus* (GN6) | isolated from aborted yak fetus | Li et al., 2017 |
|  |  |  |
| Plasmids |  |  |
| pBV220 | λpR/pL-CI857 temperature control sensitive system expression vector | Our lab |
| pUC57 Kan | Cloning vectors containing the pUC replicon | Our lab |
| pACYCDuet-1 | Expression vectors containing two multiple cloning sites | Our lab |
| pUC-E | Contains the phage PhiX174-E gene | This study |
| pUC-InpN | Contains the InpN gene | This study |
| pUC-MBP | Contains the MBP gene | This study |
| pUC-SbsA | Contains the SbsA gene | This study |
| pET28a-MOMP | Contains the MOMP gene | This study |
| pET28a-GFP | Contains the GFP gene | This study |
| pET28a-Hsp70 | Contains the Hsp70 gene | This study |
|  |  |  |
| Cells |  |  |
| L929 |  | Our lab |

**Supplementary Table 2** Primers used in this study

| Primer name | Primer sequence (5'−3') | Restriction site |
| --- | --- | --- |
| E-F | GTT ***GGATCC*** ATGGTACGCTGGACTTTGTG | BamH I |
| E-R | TAC ***CTGCAG*** TCACTCCTTCCGCACGTAA | Pst I |
| WK-E-F | TTT ***GGTACC*** GACCAGAACACCTTGCCGAT | Kpn I |
| WK-E-R | ACG ***GCATGCG*** TAGAAACGCAAAAAGGCCA | Sph I |
| mut-F | AAATATCTAACACCGCGCGTGTTGGCTATTTTACCTCTGG |  |
| mut-R | CCAGAGGTAAAATAGCCAACACGCGCGGTGTTAGATATTT |  |
| MOMP-F1 | CCG ***CCATGG*** TGGGAAGGTGCCTCTGGCGA | NcoI |
| MOMP-F2 | CCG ***GTCGAC*** TGGGAAGGTGCCTCTGGCGA | Sal I |
| MOMP-F3 | CCG ***CATATG*** TGGGAAGGTGCCTCTGGCGA | Nde I |
| MOMP-R1 | TTT ***GCGGCCGC*** TTAAAAACGAAACTGTGC | Not I |
| MOMP-R2 | TTT ***CTCGAG*** TTAAAAACGAAACTGTGC | Xho I |
| GFP-F1 | AAT ***GGATCC*** GAGCAAGGGCGAGGAGCTGTTCA | BamH I |
| GFP-F2 | AAT ***GTCGAC*** GTGAGCAAGGGCGAGGAGCTGTT | Sal I |
| GFP-R | TTT ***GCGGCCGC*** TTACTTGTACAGCTCGTCCATGCCG | Not I |
| Hsp70-F1 | GTA ***GGATCC*** AGTGAAAGACGTACTGCTGCTGC | BamH I |
| Hsp70-F2 | AAT ***GTCGAC*** GAAGTGAAAGACGTACTGCTGC | Sal I |
| Hsp70-R | AAT ***GCGGCCGC*** TTATTTTGCTTCGCGACCGT | Not I |
| E'-F1 | GTT ***GGATCC*** ATGGTACGCTGGACTTTGTG | BamH I |
| E'-F2 | CTT ***CCATGG*** GCATGGTACGCTGGACTTT | NcoI |
| E'-R | TTT ***GTCGAC*** GACGCTCGACGCCATTAA | Sal I |
| InpN-F | AATG ***CCATGG*** GCAATCTGGACAAAGCTCTGGT | NcoI |
| InpN -R | ATAT ***GTCGAC*** CTGCACATTCTGCGGCGTCGT | Sal I |
| MBP-F | GGG ***CCATGG*** GCATGAAAATCGAAGAAGGTA | NcoI |
| MBP-R | TTT ***GTCGAC*** AGTCTGCGCGTCTTTCAG | Sal I |
| SbsA-F | GGC ***CCATGG*** ATAGGAAAAAAGCTGTGAA | NcoI |
| SbsA-R | GGG ***GTCGAC*** CTTAATATAGAATGTAATAG | Sal I |

Note: italics indicate restriction enzyme sites.
